# PReS-FINAL-2241: Cases of cryopyrin-associated periodic syndromes (CAPS) in one single rheumatologic center of Russia

**DOI:** 10.1186/1546-0096-11-S2-P231

**Published:** 2013-12-05

**Authors:** SO Salugina, E Fedorov, N Kuzmina, E Zaharova

**Affiliations:** 1 Children, Federal State Budgetary Institution "Research Institute of Rheumatology" under the Russian Academy of Medical Sciences, Moscow, Russian Federation; 2 Federal State Budgetary Institution Research Centre for Medical Genetics under the Russian Academy of Medical Sciences, Moscow, Russian Federation

## Introduction

Cryopyrin-associated periodic syndromes (CAPS) are the rare hereditary autoinflammatory diseases. CAPS include three similar conditions are distinguished which lie along a phenotypical continuum with increasing levels of severity: familial cold autoinflammatory syndrome (FCAS), Muckle-Wells syndrome (MWS) and CINCA/NOMID. Distinguishing features include cutaneous, neurological, ophthalmologic and rheumatologic manifestations. CAPS caused by the mutation of the NLRP3 (CIAS1) gene coding for cryopyrin.

## Objectives

to investigate clinical and genetic characteristics of the pts with CAPS in single Federal rheumatologic center of Russia.

## Methods

**7 **patients with CAPS were followed at our centre for 5 years since 2007. There are 3 boys, 4 girls, aged 2-16 years (mean 7,9 ± 5,2 years) with disease's duration from 2 to 15 years. In 2 boys was diagnosed CINCA/NOMID, 5 - MWS. All patients were submitted to routine rheumatology examination and laboratory parameters, genetic testing in all pts was performed in research centre for medical genetics in Moscow.

## Results

Age of the first manifestation was from the birth to 9 years. All pts have had persistant or recurrent episodes fever, cutaneus rash (urticaria-like rash or maculo papular). 5 of 7 pts have had ocular manifestations: 3- conjunctivitis, 4-uveitis. 2 - no ocular abnormalities. Sensoryneural hearing loss was diagnosed in 2 boys with CINCA/NOMID and in 2 girls with MWS. Joint involvement were observed in 6 of 7 (artralgias/non-destructive arthritis - 5, bone deformities - 1 and growth retardation in 2 boys with CINCA/NOMID). Central nervous system damage (hydrocephaly, atrophy on brain, mental retardation) was in 3 pts (2 - CINCA/NOMID, 1- MWS). The laboratory findings included an elevation of acute phase reactants: ESR, neutrophil leukocytosis, C-reactive proteine rise, anemia. ANA and RF were negative. A genetic analysis on all pts showed a new mutations (heterozygous) in the NLRP3 gene: G569R, Met406Thr, Thr436Ile, Y441H, P350L, Thr438Ile, Gly455Term. No pts with AA amyloidosis and renal failure.

## Conclusion

CAPS (CINCA/NOMID and MWS) are rare hereditary auto-inflammatory diseases but it can occur in Russia. CINCA/NOMID pts have the most serious clinical manifestations and prognosis. MWS pts more often have recurrent episodes fever, cutaneous rash, conjunctivitis. Pts with CAPS need early anti IL-1 treatment for the successful in suppressing inflammation, reduction in the number and duration attacks, for prevention of amyloidosis and improvement of life prognosis.

## Disclosure of interest

None declared.

